# Heart Failure and Osteoporosis: Shared Challenges in the Aging Population

**DOI:** 10.3390/jcdd12020069

**Published:** 2025-02-13

**Authors:** Roberto Spoladore, Claudio Mario Ciampi, Paolo Ossola, Andrea Sultana, Luigi Paolo Spreafico, Andrea Farina, Gabriele Fragasso

**Affiliations:** 1 Heart Failure Clinic, Division of Cardiology, Alessandro Manzoni Hospital, ASST Lecco, 23900 Lecco, Italy; a.farina@asst-lecco.it; 2 Health Science Department, University of Milan Bicocca, 20126 Milan, Italy; claudiomariociampi@gmail.com (C.M.C.); ossola.paul@gmail.com (P.O.); a.sultana@campus.unimib.it (A.S.); 3 Orthopedics and Traumatology Unit, San Paolo University Hospital, 20142 Milan, Italy; lpspreafico@gmail.com; 4 Heart Failure Clinic, Division of Cardiology, IRCCS San Raffaele University Hospital, 20132 Milan, Italy; fragasso.gabriele@hsr.it

**Keywords:** osteoporosis, heart failure, bone metabolism, elderly age

## Abstract

In clinical practice, heart failure (HF) and osteoporosis (OP) are commonly paired conditions. This association is particularly relevant in patients over the age of 50, among whom its prevalence increases dramatically with every decade of life. This can be especially impactful since patient prognosis when facing both conditions is poorer than that of each disease alone. Clinical studies suggest that prior fractures increase the risk for heart failure hospitalization and, conversely, an episode of heart failure increases the risk of subsequent fractures. In other words, the relationship between osteoporosis and heart failure seems to be two-way, meaning that each condition may influence or contribute to the development of the other. However, the details of the pathophysiological relationship between HF and OP have yet to be revealed. The two conditions share multiple pathological mechanisms that seem to be intertwined. Patients affected by OP are more prone to develop HF because of vitamin D deficiency, elevation of parathyroid hormone (PTH) plasma levels, and increased Fibroblast Growth Factor 23 (FGF-23) activity. On the other hand, HF patients are more prone to develop OP and pathological fractures because of low vitamin D level, high PTH, chronic renal failure, alteration of renin–angiotensin–aldosterone system, reduced testosterone level, and metabolic effects derived from commonly used medications. Considering the increasingly aging worldwide population, clinicians can expect to see more often an overlap between these two conditions. Thus, it becomes crucial to recognize how HF and OP mutually influence the patient’s clinical condition. Clinicians attending these patients should utilize an integrated approach and, in order to improve prognosis, aim for early diagnosis and treatment initiation. The aim of this paper is to perform a review of the common pathophysiological mechanisms of OP and HF and identify potentially new treatment targets.

## 1. Introduction

Heart failure (HF) and osteoporosis (OP) are aging-associated diseases with shared risk factors. The prevalence of HF is below 2% in individuals under 60, rising above 6% in the following two decades and reaching 12% by age 80 [1,2]. A similar picture emerges with OP, as the risk of hip fractures more than triples from 50 to 80 years of age [3,4]. Another commonality between HF and OP is their many shared etiologic factors such as diabetes, dyslipidemia, smoking, hypertension, reduced glomerular filtration rate, decreased exercise tolerance, altered vitamin D status, increased parathyroid hormone (PTH) secretion, poor self-reported health status, and estrogen deficiency (in post-menopausal women). In addition, the medications prescribed to these elderly patients for their heart conditions put them at risk for falls with subsequent fractures. Furthermore, they are more at risk than young people for the maladaptive effects of oxidative stress, inflammation, and hyperhomocysteinemia, which can induce maladaptive remodeling of the heart and bones. We have yet to determine if the association between HF and OP is the result of two distinct pathological entities in the elderly or if there is a more reciprocal causality. This paper will address the functional association between OP and HF and discuss the possibility of an overlapping pathogenesis [5,6]. The latter hypothesis is supported by the fact that bone metabolism and vascular physiology share a number of intriguing common features [7]. In atherosclerosis, the calcification of arterial tissue is not merely a passive process of calcium absorption but a highly organized and active process determined by mechanisms similar to those involved in bone mineralization [8]. In other words, endothelial dysfunction and coronary calcifications both play a role in starting and advancing atherosclerotic lesions, and at the same time, they also contribute to the decrease in bone mineral density (BMD) and the increase in bone turnover [9].

The diagnosis of cardiovascular diseases, either myocardial infarction, HF, or stroke, is related to the subsequent risk of osteoporotic fracture. The relationship of cardiovascular disease with fractures seems strongest for HF and hip fracture [10]. When assessing the temporal sequence of the occurrence of these two conditions, prior fracture was associated with HF at least as strongly as HF associated with subsequent fracture. In both instances, the correlation was most evident for hip fractures rather than other types of fractures [11]. In fact, the detection of low volumetric bone mineral density (BMD) by High Resolution Peripheral Quantitative Tomography (HRpQCT) has been shown to be linked to increased cardiovascular disease morbidity and mortality, particularly ischemic heart disease [12].

In this context, recent epidemiological studies have revealed two seemingly opposite scenarios: a substantial increase in osteoporotic hip fractures associated with HF [13], suggesting this condition as a possible cause of the fracture and, on the other hand, a substantial increase in HF incidence at follow-up in a healthy cohort with lower BMD—suggesting lower BMD as a risk factor for the development of HF [14]. In other words, the connection between OP and HF is mutual, with each condition potentially influencing or worsening the other. As discussed in a recent editorial [15], this link raises a myriad of new questions that may yield insights into the prevention of both conditions and open a new area of cardiovascular/bone biology research.

The aim of this paper is to perform a review of the common pathophysiological mechanisms of OP and HF and to identify possible new treatment targets.

## 2. Pathophysiology of Osteoporosis and Common Risk Factors

The pathophysiological relationship between OP and HF is not completely clear. OP is characterized by reduced bone mass per unit volume of anatomical bone, which predisposes individuals to an increased risk of fractures [16]. Reduced bone strength and increased fragility derive from disrupted bone micro-architecture, porosity of the cortical compartment, altered bone macro-architecture, and decreased viability of osteocytes. Genome-wide association studies have demonstrated a genetic basis for the development of OP, identifying genes involved in the Receptor Activator of Nuclear Factor Kappa-B (RANK)/Receptor Activator of Nuclear Factor Kappa-B Ligand (RANKL)/osteoprotegerin (OPG) axis and Wnt/Wnt-β-catenin signaling [17]. Similarly, the basis for the association between reduced bone strength and risk of HF was identified in a common genetic predisposition in a twin study [18].

The bone remodeling process is choreographed by osteocytes, which are derived from osteoblasts entrapped in the mineralized matrix [19]. Disruption of osteocytes leads to decreased mechanotransduction and other essential functions related to mechanical loading. Whether due to such direct damage or simply through the normal wear and tear acquired with age, osteocytes eventually lose their ability to repress bone resorption and start to express osteoclast-stimulating factors such as RANKL, leading to bone loss. Finally, they regulate bone formation, modulating the production of sclerostin (Wnt antagonist) and Fibroblast Growth Factor 23 (FGF23) [20,21].

**Figure 1:**
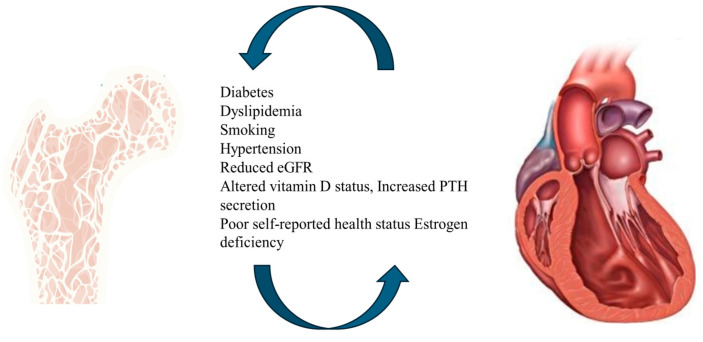
Heart failure (HF) and osteoporosis (OP) are both age-related conditions that share several risk factors. The Figure reports the common risk factors that act synergistically to create a predisposition for the development of the two diseases.

HF and OP share a number of risk factors such as advancing age, low level of 25-hydroxy-vitamin D [13,22], renal disease [23,24], diabetes [25,26], decreased exercise tolerance [27,28], and postmenopausal status [29] (Figure 1). However, the underlying mechanisms still remain unclear. One potential explanation could be the activation of the renin–angiotensin–aldosterone system (RAAS) that contributes to osteoporosis and its progression, activating osteoclasts [30,31]. Another potential mediator could be osteoprotegerin (OPG), a key regulatory protein in bone metabolism [32], found to be expressed in the heart and vasculature [33,34]. In clinical studies, serum OPG was demonstrated to be positively associated with BMD [35,36], and its expression was reported to be increased in patients with HF [37,38].

In the following two sections, we will analyze the pathophysiological relationships between HF and OP.

## 3. From Heart Failure to Osteoporosis

About half of heart transplantation candidates have osteopenia or OP, with elevated plasma levels of bone resorption markers [22]. In a recent study that evaluated an unselected cohort of patients with chronic HF, reduced bone mineral density correlated with NYHA class, peak VO_2_ consumption, and LVEF. Furthermore, patients with HF had lower bone mineral density compared to an age-matched healthy cohort. In addition, the severity of HF was an independent predictor for bone fragility [39].

According to the literature, the increased risk of OP in patients with HF is based on certain conditions that are commonly found in this sub-group of patients: oxidative stress, altered RAAS, hyperaldosteronism, renal insufficiency, increased adrenergic drive, reduced testosterone production, secondary hyperparathyroidism, hypovitaminosis D, hyperhomocysteinemia, altered IGFb1 pathway, altered FGF21 pathway and the assumption of medications for HF (Table 1).

### 3.1. Oxidative Stress

It is typically increased in patients affected by HF, and it has also been linked to decreased bone formation through enhanced osteoblast apoptosis and increased bone resorption. Osteoclast differentiation, activation, and viability are based on RANKL signaling, which requires the presence of reactive oxygen species, in particular H_2_O_2_ [40,41].

### 3.2. Altered Renin—Angiotensin—Aldosterone System

Cross-talk between Angiotensin II and RANKL pathway happens through cAMP signaling. Levels of AMP, increased by Angiotensin II, upregulate receptor activator of RANKL in osteoblasts [42] and then activate osteoclasts [30].

*Hyperaldosteronism*. Neurohormonal activation in congestive HF leads to increased aldosterone levels, which promotes oxidative stress, a pro-inflammatory phenotype, and urinary loss of Mg^2+^ and Ca^2+^, in addition to the well-known effects of sodium retention [43]. In rats receiving aldosterone by implanted minipump, plasma ionized Ca^2+^ and Mg^2+^ were reduced, resulting in bone resorption shown by reduction in BMD of tibia and femur. Despite the low plasma level concentrations, hyperaldosteronism resulted in myocyte intracellular calcium overload (“Ca^2+^ paradox”) [43]. In addition, alteration of intracellular levels of Ca^2+^ and Mg^2+^ are linked to the widespread proinflammatory status, which favors bone loss [44].

### 3.3. Renal Insufficiency

Studies in patients with HF showed an association between increased bone resorption and renal insufficiency [45,46]. This mechanism is explained by reduced calcitriol synthesis and secondary hyperparathyroidism. Moreover, FGF23 levels, which inhibit bone mineralization, increase with renal dysfunction [47].

### 3.4. Increased Adrenergic Drive

The interaction between Receptor Activator of Nuclear Factor Kappa-Β Ligand (RANKL) activation and adrenergic agonists has been explored in various studies, particularly concerning bone metabolism. Adrenergic signaling, especially through β2-adrenergic receptors (β2-AR), has been shown to influence bone remodeling by modulating RANKL expression [48,49,50]. In osteoblasts, activation of β2-AR by adrenergic agonists can upregulate RANKL expression, leading to increased osteoclastogenesis and bone resorption. For instance, a study demonstrated that dexamethasone stimulates β2-AR expression in differentiated osteoblasts, enhancing their responsiveness to adrenergic stimulation and subsequently increasing RANKL expression. This suggests that glucocorticoid-induced bone loss may be partly mediated by the heightened sensitivity of bone-forming cells to sympathetic signals [51]. Clinical studies have also examined the effects of β-adrenergic agonists and antagonists on bone metabolism. The activity of β adrenergic receptors is closely related to bone metabolism, inhibiting bone formation and reducing bone mass and bone mineral density [52]. β1 and β3 adrenergic receptors participate in bone metabolism in different ways. Selective beta-blockers promote osteogenesis, increase bone mass, contribute to antiosteoporosis, and prevent fracture, but there is not enough evidence to support nonselective beta-blockers for the clinical treatment of osteoporosis and fracture [52,53]. More interventional and observational studies are needed to confirm the antiosteoporotic effect of beta-blockers and identify a safe and effective dose range [52,53].

### 3.5. Reduced Testosterone Production

Low serum levels of testosterone and dehydroepiandrosterone (DHEA) are independently associated with low bone mass in HF patients [39]. Osteoblast activity, proliferation, and differentiation are stimulated by both testosterone and DHEA through cross-talk with androgen receptor signaling and OPG/RANKL system [54,55]. Moreover, osteoclast formation and subsequent bone resorption are inhibited by testosterone in an androgen receptor-mediated manner [55,56]. Finally, DHEA (a precursor of estrogens and androgens) reduces bone resorption and enhances bone formation by an interaction with estrogen and androgen receptors [57,58].

### 3.6. Secondary Hyperparathyroidism

The pathogenesis of increased PTH levels in HF is multifactorial: reduced cardiac output and subsequent renal impairment as well liver dysfunction leading to reduced vitamin D production; intestinal congestion jeopardizes dietary Ca^2+^ and vitamin D absorption; the use of furosemide is associated with increased divalent cation excretion and hyperaldosteronism, both associated with secondary hyperparathyroidism [22,59]. In addition, there is growing evidence that PTH is a positive cardiac inotrope and leads to an increase in heart rate and coronary blood flow [60,61]. From this perspective, the development of secondary hyperparathyroidism in chronic HF may be considered as a compensatory mechanism [60]. We also know that PTH levels are higher in patients with decompensated acute HF [62]. Increased PTH levels enhance bone catabolism [63], especially of cortical bones as the femur. A recent study demonstrated that reduced BMD in people affected by HF had a prognostic effect in predicting mortality. This also suggests that T-scores and Z-scores can also serve as prognostic tools in such patients [59].

### 3.7. Hypovitaminosis D

About 90% of patients with HF have hypovitaminosis D [64], even those living in sunny climates [65]. Such patients are usually housebound and have reduced solar exposure with subsequently reduced vitamin D synthesis. Moreover, right ventricular failure may result in liver dysfunction associated with reduced 25(OH) vitamin D production and intestinal congestion, which leads to reduced absorption of dietary vitamin D [66]. Finally, 1-25(OH)_2_ vitamin D production is reduced with worsening renal function [21].

### 3.8. Hyperhomocysteinemia

It is a well-known condition associated with thrombotic and premature atherosclerotic cardiovascular disease [67]. A meta-analysis showed that high plasma levels of homocysteine were an independent risk factor for fractures. This fits with the pathophysiological concept that cross-linking of type I collagen, vital for bone matrix formation, is inhibited by homocysteine [68]. Type I collagen is the most abundant form of collagen in the human body, accounting for approximately 90% of the total collagen. It is essential for the structural integrity of tissues such as skin, bones, tendons, and ligaments. Its structure is characterized by a triple helix composed of two pro-alpha1(I) chains and one pro-alpha2(I) chain, encoded by the COL1A1 and COL1A2 genes, respectively. A study highlighted that N-homocysteinylation of collagen in mice deficient in cystathionine β-synthase (CBS), a model for hyperhomocysteinemia, impairs the formation of collagen cross-links. This phenomenon may contribute to connective tissue abnormalities observed in hyperhomocysteinemia conditions [69]

### 3.9. Altered FGF-21 and IGFb1 Pathways

Expression of FGF21 in myocytes is regulated by the Sirt1-PPARalfa pathway and is increased in response to various cardiac stressors. It may function in an autocrine manner, protecting from cardiac hypertrophy through PGC1alfa expression, which is involved in the control of oxidative stress and energy metabolism [70]. If it has positive effects in the failing heart, it is associated with bone loss and skeletal fragility [71]. Furthermore, FGF21 induces liver synthesis and secretion of IGFb1, which promotes osteoclast differentiation and potentiates the RANKL signaling pathway, resulting in bone resorption [72,73].

### 3.10. Medications for Heart Failure

Loop diuretics are associated with reduced hip BMD in men [74] and total fractures in women [75], while thiazide diuretics, spironolactone, and beta-blockers may be protective from this condition [76,77]. Loop diuretics may increase calcium excretion and lead to secondary hyperparathyroidism, while thiazide diuretics have the opposite effect. In addition, spironolactone reduces aldosterone levels (see hyperaldosteronism) and increases potassium stores, which are linked to bone strength increase [78,79]. Beta-blockers seem to inhibit epinephrine-mediated activation of RANKL and, therefore, bone resorption [80,81].

Sacubitril/valsartan, a combination of a neprilysin inhibitor (sacubitril) and an angiotensin receptor blocker (valsartan), is a cornerstone in HF therapy. The impact of this drug on bone metabolism and OP has not been thoroughly researched. Both components work together to reduce blood pressure, alleviate HF symptoms, and improve outcomes in patients with HF with reduced left ventricular ejection fraction [82]. However, recent studies suggest that sacubitril/valsartan may have indirect effects on bone health. Angiotensin II, a key mediator in the renin–angiotensin system, plays a role in bone remodeling by stimulating osteoclast activity, which promotes bone resorption [83]. By inhibiting this pathway, valsartan may theoretically reduce osteoclast activity and protect against bone loss. Similarly, neprilysin inhibition by sacubitril may reduce the degradation of natriuretic peptides, which have been shown to influence bone formation. Although preclinical studies have indicated beneficial effects of sacubitril/valsartan on bone turnover, more clinical research is needed to fully understand its impact on OP [84,85].

Sodium-glucose co-transporter 2 (SGLT2) inhibitors, such as empagliflozin and dapagliflozin, are a class of drugs primarily used to manage type 2 diabetes, HF, and chronic kidney disease [86,87]. Emerging evidence suggests that these medications may have beneficial effects on bone health, potentially impacting OP. SGLT2 inhibitors work by inhibiting the resorption of glucose in the kidneys, leading to increased glucose excretion. Recent studies have indicated that SGLT2 inhibitors may improve bone mineral density and reduce the risk of fractures [88,89]. The mechanisms behind these effects are not yet fully understood, but it is believed that SGLT2 inhibition may enhance bone formation by reducing serum phosphate levels and increasing the production of FGF-23. Additionally, the reduction in blood glucose and the associated decrease in insulin resistance could positively influence bone metabolism. However, while some studies suggest a protective effect on bone health, other research has raised concerns about an increased risk of fractures, particularly in patients with kidney disease or those receiving long-term treatment. Further large-scale, long-term clinical trials are needed to clarify the full impact of SGLT2 inhibitors on bone health and OP risk [90,91].

## 4. From Osteoporosis to Heart Failure

A recent prospective population-based study recruited 25.639 men and women aged 39–79 years in an apparent state of good health; authors showed for the first time that BMD, with or without established OP, was inversely associated with risk of HF [14]. After adjusting for many risk factors, for each 1 standard deviation increase in ultrasound bone attenuation, the risk of HF decreased by 23%. This study suggests that screening for OP might identify people who are at risk for incident HF and in whom early initiation of treatment might be helpful.

As well as shared risk factors, there are also multiple pathophysiological mechanisms that might explain this association.

### 4.1. Hypovitaminosis D

We have seen that HF is associated with a state of hypovitaminosis D. It is also evident that low levels of vitamin D are associated with an increased risk of developing chronic HF [92]. In addition, vitamin D may modulate the severity of HF [93]. Low levels of vitamin D could lead to cardiac dysfunction and adverse remodeling through numerous mechanisms: interference with Ca^2+^ transport and subsequent intracellular calcium overload [94]; contribution to cardiomyocyte hypertrophy, interstitial fibrosis and inflammation [95,96]; as well as the enhancement of RAAS activity [97]. Consistently, vitamin D receptor knock-out mice show increased RAAS activity, which leads to hypertension and cardiac hypertrophy. 1,25-Dihydroxyvitamin D3 is a negative endocrine regulator of the renin–angiotensin system [98]. Thus, we can consider vitamin D as a protective agent against HF. In an experimental study on animals, Simpson et al. noticed that mice with a knock-out for vitamin D receptors were prone to develop cardiac hypertrophy [99]. Myocardial contractility has been shown to increase after administration of vitamin D in animal studies [100].

### 4.2. Secondary Hyperparathyroidism

Regardless of its plasma levels, PTH promotes Ca^2+^ entry into myocytes, ensuing intracellular Ca^2+^ overloading [101]. Therefore, high levels of PTH are related to calcium overload [102] and subsequent oxidative stress, impaired electromechanical coupling, mitochondrial dysfunction, and increased cellular apoptosis [103]. In addition, primary hyperparathyroidism is a risk factor for overt atherosclerosis and, thus, for acute coronary syndromes and post-ischemic cardiac dysfunction [104]. In a cohort of over 9000 middle-aged predominantly Caucasian patients, PTH levels > 75 pg/mL were associated with a higher risk of cardiovascular events [105]. In addition, serum PTH levels > 65 pg/mL resulted in a 50% increase in HF risk in the Multi-Ethnic Study of Atherosclerosis, a US population study on over 6400 participants free of cardiovascular disease [106].

### 4.3. Altered FGF23 Pathway

Fibroblast Growth Factor 23 (FGF23) is a member of the FGF19 subfamily, and it is mainly produced by osteocytes. It is a phosphatidic hormone and also an inhibitor of bone mineralization [107]; high FGF23 levels are associated with hypophosphatemic rickets [108]. In an experimental study utilizing a mice model of chronic kidney disease (CKD), elevations of circulating FGF23 are associated with adverse cardiovascular outcomes, progression of renal failure in CKD and renal allograft loss; moreover in CKD mice model, FGF23 circulating concentration is one of the strongest predictors of mortality and adverse cardiovascular outcomes, suggesting that in this population FGF23 is related to direct cardiovascular toxicity [109]. Inflammation is a possible explanation for FGF23-induced toxicity [110]. FGF23 can also directly induce cardiomyocyte hypertrophy [111] by regulating intracellular Ca^2+^ level: it opens L-type Ca^2+^ channels on cardiomyocytes’ extracellular membrane and increases cardiac contractility through calcium-induced calcium release (CICR) mechanism. Similarly to other neurohormonal systems, long-standing compensatory activation of FGF23 may determine a maladaptive remodeling in HF, leading to worsening of left ventricular function [112]. Lastly, a recent study recognized FGF23 as a risk factor for atherosclerosis and showed that it was independently associated with carotid plaques and total carotid plaque area (TCPA) [113].

## 5. Therapy

**Figure 2:**
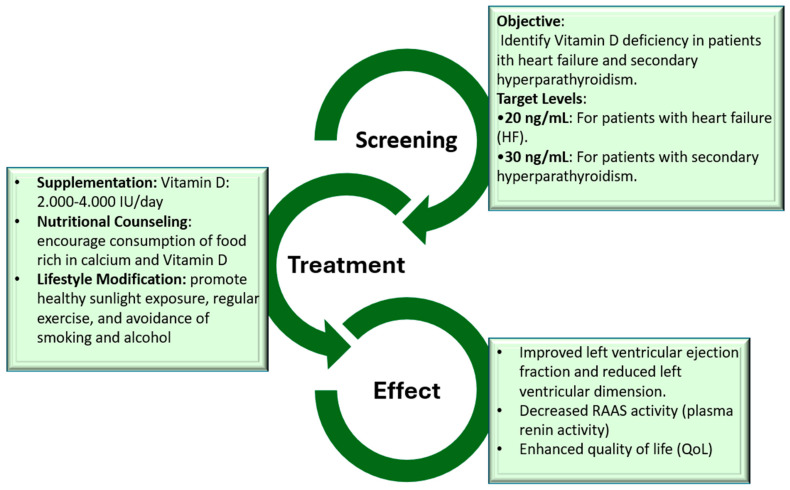
Key steps in vitamin D deficiency screening and management.

On the basis of the aforementioned pathophysiological connections and the multitude of common risk factors, a combined treatment for patients affected by both OP and HF should be the goal. However, treatment of OP could influence the clinical course of HF and vice versa. Since the effects of HF drugs on the course of OP have already been analyzed, the potential effects of OP treatment on the prognosis of patients with HF will now be discussed (Figure 2).

Screening for vitamin D deficiency is advocated in most patients. According to current guidelines, vitamin D goals of >20 ng/mL in most patients with HF and >30 ng/mL in those with secondary hyperparathyroidism seem to be appropriate [114].

In 2006, Schleithoff and his team performed the first randomized controlled trial (RCT) on 93 subjects who received either 50 μg vitamin D3 with calcium per day or placebo with calcium. At the end of the 9-month follow-up period, a non-significant improvement of LV function was observed in the active arm of the study [115]. More recently, Boxer et al. demonstrated improvements in serum aldosterone and quality of life in patients treated with vitamin D supplementation [116]. Schroten et al. showed a reduction in the activation of RAAS, in terms of plasma renin activity, in patients who were supplemented with vitamin D [97]. An improvement of LVEF in supplemented patients was first found by Dalbeni et al. in 2014 [117]. A recent randomized double-blind placebo-controlled study (VINDICATE study) (Effects of Vitamin D on Cardiac Function in Patients With Chronic HF: The VINDICATE Study) showed that an oral non-calcium-based daily supplement of 4.000 IU of vitamin D3 administered for 12 months in patients with chronic HF, already treated with optimal medical therapy, was associated with improvement in left ventricular ejection fraction and a reduction of left ventricular dimensions and volumes [118]. However, the study failed to show an improvement in 6MWT distance (primary outcome). Such a result can be partially explained by the high variability in the walking distance at baseline of the enrolled patients.

Over the years, some studies have reported controversial conclusions. However, in 2018, Zhao et al. performed a meta-analysis including seven major RCTs on the effect of vitamin D on ventricular function in the context of HF [119]. The study showed that the supplementation of vitamin D increased LVEF and reduced LV volumes, producing a significant reverse remodeling in LV.

However, more data are needed to show convincing improvement in CV clinical outcomes with vitamin D replacement. Nevertheless, while several reports have shown improved LV performance, no study has reported adverse effects from vitamin D supplementation.

Vitamin D replacement is economical and safe for patients with HF. Therefore, our recommendation is to screen for and correct vitamin D deficiency in all patients diagnosed with HF. All patients presenting with low plasma levels of vitamin D (<30 ng/mL) should receive dietary supplementation (calcium), nutritional counselling (to promote eating foods rich in calcium and vitamin D), and lifestyle counselling (avoidance of alcohol and smoking, exercise, healthy sunlight exposure). Vitamin D levels should be reassessed routinely in order to tailor the therapy.

## 6. Future Research and Perspectives

Current evidence suggests a complex relationship between osteoporosis and heart failure, with overlapping pathophysiological mechanisms involving inflammation, hormonal imbalances, and impaired physical activity. Both conditions are prevalent in the aging population, yet the exact interplay remains controversial. On one hand, chronic inflammation and oxidative stress, common in HF, can contribute to bone resorption and decreased bone density, while reduced mobility in heart failure patients further exacerbates bone loss [120]. On the other hand, as already described, certain HF treatments, such as diuretics, can lead to electrolyte imbalances, which may negatively affect bone health. Moreover, the use of certain medications, including beta-blockers and ACE inhibitors, has been shown to have both positive and negative effects on bone metabolism, adding to the complexity of managing these conditions concurrently [121]. Controversy remains regarding the best therapeutic approach: should treatments for osteoporosis be prioritized in HF patients, or is managing heart failure symptoms more critical? Further research is needed to clarify these interactions and to develop comprehensive, patient-specific management strategies.

In older adults, OP and HF are frequently co-occurring conditions that add extra challenges to their clinical management. The intricate relationship between osteoporosis and heart failure highlights the complex interplay between bone health and cardiovascular function. While current research has provided valuable insights into shared risk factors and underlying pathophysiological mechanisms, much remains to be explored. Future studies should focus on several key areas to improve our understanding and management of these coexisting conditions. First, there is a need for in-depth research into the molecular and cellular pathways that link osteoporosis with heart failure. Understanding how chronic heart failure may accelerate bone loss and, conversely, how osteoporosis could exacerbate cardiovascular dysfunction is crucial. Investigating the role of systemic inflammation, oxidative stress, and altered hormonal regulation could provide insights into shared mechanisms that affect both bone and cardiovascular systems. Moreover, more large-scale longitudinal studies are necessary to identify the precise relationship between osteoporosis and heart failure across different populations, particularly in those with comorbidities, such as diabetes or chronic kidney disease. Additionally, randomized controlled trials are critical to assess the effectiveness of integrated treatment strategies that address both bone health and cardiovascular function. For instance, exploring the potential benefits of osteoporosis treatments in patients with heart failure could yield important clinical guidance. It would also be beneficial to examine how heart failure management strategies, including device-based therapies like cardiac resynchronization or heart transplant, might influence bone density or fracture risk. Finally, interdisciplinary collaboration between cardiologists, endocrinologists, rheumatologists, and geriatricians will be vital to developing comprehensive management protocols. These protocols should not only focus on the optimal treatment of each individual condition but also consider the overall well-being of patients, promoting a holistic approach to care.

## 7. Conclusions

Recent epidemiological research has shown that heart failure and osteoporosis are conditions linked through bilateral associations and common underlying mechanisms. Both conditions lead to significant morbidity and mortality. Clinicians attending these patients should utilize an integrated approach and aim for the earliest diagnosis and treatment initiation to improve the prognosis. Early, combined treatment may improve the prognosis for both conditions. Recent studies demonstrate that vitamin D supplementation can reverse LV remodeling in patients with HF. Awareness of the functional association between HF and OP is increasing, but many gaps in our knowledge remain. By expanding our understanding in these areas, we can significantly improve patient outcomes and quality of life for individuals affected by both osteoporosis and heart failure.

## Figures and Tables

**Table 1:** Shared pathophysiological mechanisms between heart failure and osteoporosis.

**Vitamin D deficiency**	Worsens HF and increases cardiac remodeling.
**Elevated PTH**	Promotes calcium overload in heart cells, exacerbating HF.
**Altered FGF23 pathway**	Linked to bone loss and adverse heart outcomes, including HF.
**FGF21 and IGFBP1 pathways**	FGF21 protects the heart but contributes to bone loss.
**Oxidative stress**	Correlates with left ventricular dysfunction and promotes bone loss by increasing osteoclast activity.
**RAAS activation**	Angiotensin II promotes adverse cardiac remodeling and bone resorption via RANKL activation.
**Adrenergic drive**	Contributes to the worsening of HF and stimulates osteoclast activity.
**Heart Failure medications**	Loop diuretics decrease BMD; beta-blockers and thiazides may be protective.

HF: Heart failure, PTH: Parathyroid hormone, RANKL: Receptor activator of nuclear factor kappa-B ligand, FGF23: Fibroblast growth factor 23, FGF21: Fibroblast growth factor 21, IGFBP1: Insulin like growth factor binding protein 1, RAAS: Renin angiotensin aldosterone system, BMD: Bone mineral density.
